# Biomimetic Dual-Sensing Bone Scaffolds: Characterization and In Vitro Evaluation Under Dynamic Culturing Conditions

**DOI:** 10.3390/biomimetics10090598

**Published:** 2025-09-08

**Authors:** Damion T. Dixon, Erika N. Landree, Cheryl T. Gomillion

**Affiliations:** 1 School of Environmental, Civil, Agricultural and Mechanical Engineering, University of Georgia, Athens, GA 30602, USA; damion@uga.edu; 2 School of Chemical, Materials and Biomedical Engineering, University of Georgia, Athens, GA 30602, USA

**Keywords:** demineralized bone matrix, ultrasound, electrical stimulation, 3D printing, biomimetic bone scaffolds, osteogenic differentiation, bone tissue engineering

## Abstract

The regeneration of large segmental bone defects remains a significant challenge. While electrical stimulation has demonstrated the potential to accelerate bone healing, clinical translation has been hindered by the lack of safe, localized delivery methods. In this study, we present a novel strategy combining piezoelectric and electrically conductive polymers with allograft demineralized bones to create stimuli-responsive, biologically relevant scaffolds via pneumatic 3D printing. These scaffolds exhibit enhanced piezoelectric potential and tunable electrical properties, enabling both electrical and mechanical stimulation of cells (without external stimulators). Under dynamic culturing conditions (i.e., ultrasound stimulation), human bone marrow-derived mesenchymal stromal cells cultured on these scaffolds displayed significantly elevated osteogenic protein expression (i.e., alkaline phosphatase and osteocalcin) and mineralization (confirmed via xylenol orange mineral staining) after two weeks. This work introduces a bioinspired, printable ink in conjunction with a simple fabrication approach for creating dual-responsive scaffolds with high potential for functional bone tissue regeneration.

## 1. Introduction

Bone tissue engineering focuses on developing therapeutic strategies for the repair of complex fractures. While bone exhibits intrinsic self-regeneration capabilities, critical-sized defects exceed this natural healing potential and require external interventions to restore tissue function and integrity [1,2]. Currently, autografts are the clinical gold standard for bone repair but are limited by availability and high associated costs [3]. Allografts provide a secondary option and are available in various forms, such as demineralized bone matrix (DBM). When compared to autografts, these donor tissues present a risk for immune rejection; however, this can be mitigated through processes such as decellularization (i.e., removal of cellular components) [4]. Due to the shortcomings of natural tissue grafts, there is a growing demand for novel and effective bone regenerative strategies [5]. Recent advancements in bone tissue engineering, particularly the development of biomimetic synthetic scaffolds, present a promising alternative for the repair of critical-sized bone defects and hold the potential to alleviate the clinical burden associated with natural tissue grafts.

Bone remodeling and regeneration are complex, highly regulated processes involving a coordinated interplay of cells, signaling molecules, and extracellular matrix components to facilitate bone healing [6,7]. To effectively participate in these processes and restore functional bone integrity, engineered constructs must closely replicate the physical and biological properties of native bone tissue [8,9]. A wide array of biomaterials, including naturally derived matrices, synthetic polymers, hydrogels, and bioactive small molecules, has been used to create bone scaffolds. In particular, synthetic polymers (e.g., polycaprolactone, PCL), due to their mechanical tunability and ease of fabrication, are frequently utilized; however, they lack bioactivity, necessitating strategies to improve their biological performance. Growth factors have been instrumental in this regard, boosting the osteoinductive properties of synthetic scaffolds [10]. Nevertheless, concerns related to their potential cytotoxicity at high concentrations or uncontrolled release have prompted ongoing efforts to develop new methods of functionalizing synthetic scaffolds.

The incorporation of electrically conductive materials to enhance scaffold bioactivity and promote positive cellular responses has been increasingly studied [11]. In parallel, external electrical stimulation (ES) has emerged as a promising modality for modulating cell behavior, offering greater control over growth factors, and demonstrating favorable outcomes in the regeneration of various tissues, particularly bone [12,13,14]. We have previously reported on the synergistic effects of combining conductive bone scaffolds and ES for enhanced in vitro mineralization of murine preosteoblasts (MC3T3-E1), highlighting this complementary strategy for bone regeneration [15]. More recently, we have developed 3D-printed electrically conductive composite scaffolds containing DBM, in conjunction with ES, as biomimetic hybrid bone scaffolds [16]. To date, there have been numerous ES devices developed for the augmentation of bone growth, with their specific ES mechanisms described elsewhere [17]. Despite the promising potential of ES, particularly in relation to in vitro mineralization, clinical translation has continued to prove less successful. Nicksic et al. suggest that a potential hinderance is related to inconsistencies in ES regimen (frequency, duration, voltage, etc.) and incomplete device specification reporting, leading to a reduction in efficacy when translated to larger models (e.g., large animals). Transcutaneous ES is further limited due to signal attenuation related to tissue thickness (or possible increase resistivity), whereas implantable ES systems are often associated with significant drawbacks, including the use of non-biodegradable and potentially cytotoxic batteries, as well as the requirement for secondary surgical procedures for device retrieval [18]. Consequently, the clinical utility of external ES is constrained when integrated with current regenerative strategies.

An alternative strategy to enhance the bioactivity of an implantable scaffold involves the use of “smart” biomaterials that are capable of responding to or generating physical stimuli (e.g., electrical or mechanical). Piezoelectric materials can generate electrical charges in response to mechanical stresses, enabling them to serve as self-powered stimulatory platforms. These materials harness physiological movements or externally applied mechanical inputs (e.g., vibrations) to induce localized electric fields that can promote osteogenic differentiation. Notably, native bone tissue exhibits intrinsic piezoelectric properties, whereby mechanical deformation induces surface charge generation that contributes to tissue remodeling and homeostasis, in accordance with Wolff’s Law [19,20,21]. Piezoelectric scaffolds, therefore, can mimic native bones by undergoing mechanical loading to generate electrical charges that self-promote bone regeneration. In fact, there have been several recent reports of favorable bone growth on charged surfaces of piezoelectric polymers such as polyvinylidene fluoride (PVDF), confirming the usefulness of these responsive materials for bone regeneration [22,23]. Piezoelectric rods prepared from poly(L-lactic acid) (PLLA) have also been utilized at the bulk scale (i.e., in vivo fracture healing in cats) [24]. In this study, the presumed piezoelectric output resulted from the passive motions of the animals, ultimately offering no control over the ES regimen (i.e., time, duration, and amount of applied ES). Therefore, a responsive bone scaffold capable of localized, on-demand ES delivery would improve considerably over contemporary repair options.

Herein, we introduce an innovative approach for the fabrication of bioactive dual-stimulus-responsive scaffolds via pneumatic 3D printing. The prepared scaffolds provide ES directly to cells through the incorporation of piezoelectric PVDF and possess sustained bioactivity though the incorporation of allograft DBM. Combined with extracorporeal ultrasound stimulation (US), the resulting constructs function as battery-free, externally modulated electrical stimulators and osteoconductive scaffolds, offering a biomimetic platform with enhanced biological and electrical properties to support bone regeneration.

## 2. Materials and Methods

**Figure 1:**
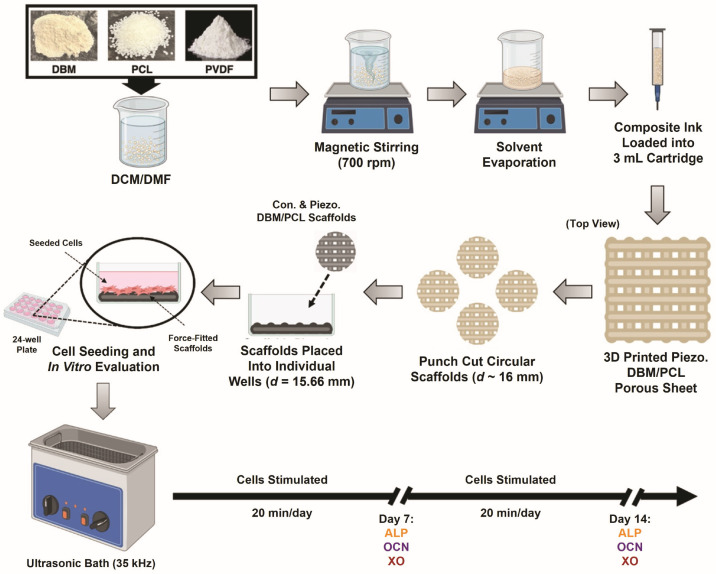
Experimental flow: Printing ink formulation, scaffold fabrication and culture setup, and ultrasound stimulation regimen. Created in BioRender. Gomillion, C. (2025) https://BioRender.com/74ltt32 (accessed on 25 August 2025).

The overall experimental flow is depicted in Figure 1, with detailed methods provided in the sections that follow:

### 2.1. Preparation of Printing Ink

All materials were used as received without further purification. Human femurs were supplied by MTF Biologics through their Non-Transplantable Tissue Program and used to prepare the DBM powder, as described previously [16]. PCL pellets (*M*_n_ = 80,000 g mol^−1^, Sigma-Aldrich, St. Louis, MO, USA) were dissolved in dichloromethane (DCM, VWR, Radnor, PA, USA) and PVDF powder (Sigma-Aldrich) was dissolved in N,N-Dimethylformamide (DMF, Fisher Scientific, Waltham, MA, USA) to produce two separate 10 wt% polymer solutions. The PCL and PVDF solutions were then combined and subjected to continuous stirring (700 rpm, at ~50 °C), resulting in a single homogenous polymer solution. Once cooled to room temperature (RT), DBM powder (<125 µm) was added, and the dual polymer mixture was vigorously stirred to form a composite ink. For this report, inks consisting of only PCL or a 50:50 wt% ratio of DBM/PCL were created and used for scaffold fabrication.

### 2.2. Scaffold Fabrication

A BIO X 3D pneumatic printer (CELLINK) was used to print inks via RT syringe (I.D. = 410 µm) extrusion. The printing pressure and speed were set to 200 kPa and 5 mm/s, respectively. Utilizing a CAD design, struts (spacing = 250 µm) were printed with each progressive layer oriented 90° relative to the underlying layer (i.e., 2 cross-hatched layers) to form thin square sheets (*l* ~ 2 cm × *h* ~ 2 cm × *t* ~ 1 mm). Following printing, scaffolds were submerged in deionized (DI) water to prevent DCM and DMF evaporation in air. The utility of this solvent exchange step is twofold: (1) it prevents the 3D geometries from collapsing/shrinking during drying and (2) it further induces high β-phase in the printed PVDF, which is already enhanced due to the directional alignment of the dipoles provided by the high shearing force of pneumatic printing. After solvent evaporation and subsequent water removal, scaffolds were cut to size (*d* ~ 16 mm × *t* ~ 800 µm) using a metal punch (VWR). To enhance scaffold conductivity (and amplify provided signals to growing cells), a combination of conductive polymers, PEDOT:PPS and PPy were grafted onto the surface of the printed composite scaffolds using a polydopamine (PDA) mediated adhesion strategy analogous to our previous report [16]. The electrical properties of coated scaffolds were acquired utilizing a Four-Point Probe System (Ossila). Sheet resistance values were calculated by placing coated scaffolds onto glass slides beneath a probe head atop the testing stage. For each scaffold, a total of 10 readings were taken and averaged (*n* = 3 per group). The average sheet resistance for scaffolds with the conductive coating (i.e., 10% in DI water) was found to be ~1.5 × 10^6^ Ω/sq.

### 2.3. Scaffold Characterization: Piezoelectric Properties and Surface Wettability

Fourier-transform infrared (FTIR) spectroscopy was performed on printed PVDF and cast PVDF films using a Nicolet 6700 FTIR spectrometer (Thermo Fisher Scientific, Waltham, MA, USA) to assess whether this fabrication method was suitable in producing a high β-phase content in PVDF (i.e., capable of producing piezoelectric scaffolds). The characteristic absorbance peaks of the α-phase at 763 cm^−1^ and β-phase at 1275 cm^−1^ were used to qualitatively investigate the existence of each crystalline phase and determine the dominant phases in the prepared samples [25,26]. The spectrometer was set to collect absorbance readings across the infrared spectra from 4000 to 700 cm^−1^. For each sample, there was a total of 256 scans collected and the spectral resolution was set to 6 cm^−1^. Quantitative measurements of the piezoelectric properties and US receiving capability of the printed PVDF and cast PVDF films were measured using a lab-made force sensor. The prepared (i.e., printed or cast) PVDF films were sandwiched between aluminum foil electrodes before being encapsulated in duct tape (Gorilla Glue Inc., Sharonville, OH, USA) to isolate the aluminum foil electrodes from air. The PVDF film powered sensor was subjected to ultrasonic waves at a 35 kHz frequency via an Ultrasonic Bath equipped with a bath temperature controller (VWR). The sensor was suspended freely (roughly centered) into the preheated (~37 °C to mimic cell culture) water bath and connected to an oscilloscope to measure the open circuit voltage.

PDA was deposited onto the surface of the printed scaffolds (prior to dynamic osteogenic culture) to anchor the conductive polymer coating and also encourage cellular adhesion. To assess scaffold hydrophilicity before and after the addition of the PDA coating, static water contact angle measurements were taken using a Contact Angle Goniometer (Ossila). Immediately (<~30 s) after a sessile DI water droplet (10 μL) was applied onto the surface of the scaffolds, photos were captured, and the water contact angle was measured (*n* = 3 per group).

### 2.4. Cell Culture and Seeding

Owing to their significant use in both preclinical and clinical models for bone healing, human bone marrow-derived mesenchymal stromal cells (hMSCs, Lonza) were utilized in this study [27,28]. Trypsin-EDTA (0.05%), Dulbecco’s Phosphate-Buffered Saline (PBS, -Ca, -Mg), Penicillin-Streptomycin (Pen-Strep), and both low-glucose (1 g/L) and high-glucose (4.5 g/L) Dulbecco’s Modified Eagle’s Medium (DMEM) were purchased from Gibco (Waltham, MA, USA). Fetal bovine serum (FBS) was purchased from R & D Systems (Minneapolis, MN, USA). Ultra-Low Attachment 24-well culture plates were purchased from Corning (Corning, NY, USA).

Cryopreserved stocks of hMSCs between passage 4–8 were revived and expanded prior to use. Cells were grown in low-glucose DMEM supplemented with 10% FBS and 1% Pen-Strep, denoted as growth medium. Once cultures presented ~90% sub-confluency, they were passaged by treatment with Trypsin-EDTA, cell pellets were formed by centrifugation (1200 rpm, 5 min), and cells resuspended in fresh growth medium. To minimize cellular attachment to the surface of culture wells and improve the cellular adhesion potential to experimental scaffolding surfaces, Ultra-Low Attachment well plates were used. For seeding, UV-sterilized (~30 min per side) scaffolds were force-fitted into individual wells (15.6 mm) and pre-wetted with growth medium. After counting, a cell suspension was created and used to seed individual, scaffold-containing wells for experiments. Well plates were stored inside of a standard 5% CO_2_ incubator at 37 °C.

### 2.5. Scaffold Biocompatibility Assessment and Composite Ink Optimization

Prior to our dynamic osteogenic culture (i.e., with US), a 12 h cellular attachment study was performed using a Cell Counting Kit-8 (CCK-8, Sigma-Aldrich) colorimetric assay to determine an ideal composition (wt% of PVDF) to add to the composite scaffolds (i.e., a preliminary assessment of scaffold biocompatibility). Cells were seeded at a density of 3 × 10^4^ cells/cm^2^ on pre-wetted scaffolds placed in 24-well plates and stored inside of an incubator. After the 12 h cellular attachment period, the CCK-8 solution was added into individual wells at roughly 10% of the culture medium volume (i.e., 50 µL of CCK-8 to 500 µL of culture medium), followed by a ~1 h incubation at 37 °C. Sample absorbance was measured at 450 nm using a Cytation 1 imaging reader (BioTek, Agilent Technologies, Santa Clara, CA, USA) and compared to the absorbance produced by cells grown on control scaffolds containing only PCL (i.e., considered to be 100% growth). Additionally, qualitative attachment was assessed using a Hoechst 33,342 (Molecular Probes, Invitrogen, Eugene, OR, USA) stain to visualize cell spreading on the various PVDF containing substrates. In this assessment, along with PCL-only scaffolds, DBM/PCL (50:50 wt%) scaffolds were utilized as a positive growth control. For PVDF containing scaffolds, the DBM ratio was maintained at 50 wt% (based on our previous observations for in vitro bone formation), while the PCL ratio was adjusted based on PVDF content (e.g., scaffolds with 10 wt% PVDF would contain 40 wt% PCL).

### 2.6. In Vitro Evaluation Under Dynamic Culturing Conditions

Based on our 12 h cellular attachment study, piezoelectric (i.e., containing PVDF) composite scaffolds were prepared for our osteogenic evaluation with the following wt% ratios: 50% DBM, 25% PCL, and 25% PVDF. Additionally, an electrically conductive variant (as per Section 2.2) of these piezoelectric composites was also evaluated to assess the potential regenerative synergy between conductive/piezoelectric scaffolding components. Herein, scaffolds will have the following designations based on their composition (by wt%): “PCL” (100% PCL), “DBM/PCL” (50% DBM, 50% PCL), “Piezo. DBM/PCL” (50% DBM, 25% PCL, 25% PVDF), and “Con. & Piezo. DBM/PCL” (50% DBM, 25% PCL, 25% PVDF with a conductive coating), further isolating the effects of each scaffolding component. All scaffold compositions were PDA-coated prior to cell seeding to encourage cellular attachment. For osteogenic differentiation, hMSCs were cultured in high-glucose DMEM supplemented with 10% FBS, 1% Pen-Strep, 50 µg/mL ascorbic acid, 10^−7^ M dexamethasone, and 10 mM β-GP, denoted as differentiation medium. Cells were seeded at a density of 8 × 10^4^ cells/cm^2^ on scaffolds placed in 24-well plates and allowed to attach inside of an incubator.

After an overnight attachment period, growth medium was exchanged with differentiation medium and US treatment began. The treatment was performed using an Ultrasonic Bath that provided ultrasound at 35 kHz, the bath temperature was held constant at 37 °C. The US regimen consisted of treatment for 20 min daily and was performed for the duration of the two-week osteogenic culture. Several measures were taken to limit the introduction of non-sterile water, from the bath, into culture wells and affecting the viability of the culture throughout this process. We first reduced the differentiation medium in each well (from 500 µL to 350 µL) to prevent spillage and reduce the chances of it getting contaminated by the water. Additionally, we sealed the entire plate utilizing a standardized method as described elsewhere [18]. In short, each plate was first taped on all 4 sides using labeling tape (to secure the lid) before being wrapped in three alternating layers of plastic wrap and duct tape. Once completely wrapped, additional small pieces of duct tape were applied to the corners of the plates to prevent water from seeping through any potential gaps. For treatment, encapsulated plates were suspended in the bath utilizing a central (to the lid of the well plate) tab created from duct tape, a clamp, a stand, and alligator clips. Plates were submerged roughly halfway into the bath and subjected to sonication for 20 min. Once US concluded, plates were unwrapped inside of a laminar flow hood, and the differentiation medium in each well was replaced with 500 µL of fresh differentiation medium. Plates were then returned to the incubator until the next US treatment. Furthermore, unstimulated cultures (i.e., those not exposed to US) were likewise removed from the CO_2_ incubator, encapsulated, and suspended in the preheated (37 °C) Ultrasonic Bath for 20 min daily (to account for any disruptions in the normal cell culture environment).

To measure osteogenic protein expression, protein samples were collected by first removing the culture medium from test wells, rinsing with PBS, and dislodging cells from scaffolds using Trypsin-EDTA. Once disassociation was confirmed by visual inspection under a microscope, the reaction was neutralized with the addition of fresh culture medium at twice the volume (i.e., 1 mL of fresh culture medium per 500 µL of Trypsin-EDTA), and the contents of each well were transferred to separate microtubes. Cell pellets were then formed by centrifugation (14,000× *g* for 15 min), after which the supernatant was removed and resuspended in Mammalian Protein Extraction Reagent (M-PER, Thermo Fisher Scientific) to extract proteins from the suspended cells. Microtubes were once again centrifuged in order to collect the supernatant (i.e., extracted protein) for further analysis. Total protein concentrations were measured using a Pierce BCA Protein Assay Kit (BCA, Thermo Fisher Scientific), as described elsewhere [15]. Total protein (Figure S1) was measured for two reasons: (1) to normalize bone specific proteins within each culture condition and (2) to assess relative viability of cells throughout the 14-day culture period. There were no significant differences among any of the groups at either time point (i.e., there was no loss in cell viability due to the US treatment).

#### 2.6.1. Alkaline Phosphatase Activity

An Alkaline Phosphatase (ALP) Assay Kit (abcam, Waltham, MA, USA) was used test for the presence of osteoblast-like cells over the course of the 14-day long culture period analogous to our previous reports [15,16]. Detailed methods for this assay are provided in the Supplementary Materials. The resulting values were normalized to total protein concentrations for each scaffold type and stimulation group.

#### 2.6.2. Osteocalcin ELISA Assay

A Human Osteocalcin ELISA Kit (#EKU06413, Biomatik, Wilmington, DE, USA) was used to measure the expression of osteocalcin, a protein preferably secreted by osteoblasts, as detailed in our earlier work [15,16]. After a series of incubation and wash steps, the plate absorbance was measured and likewise normalized as described previously. Detailed methods for this assay are also provided in the Supplementary Materials.

#### 2.6.3. Xylenol Orange Mineral Staining

To stain mineralized nodules within the different scaffolding groups, xylenol orange (XO), a nondestructive calcium-chelating fluorescent stain was used, as described previously [29]. Before imaging, the differentiation medium was replaced with sterile PBS, and the scaffolds were inverted. Calcium deposition was detected by viewing under a TRITC (shown in red) filter; the objective magnification was consistent for all images.

### 2.7. Statistical Analysis

All quantitative data was produced in triplicate (*n* = 3) and is expressed as mean ± standard deviation. Statistical analysis was performed using GraphPad Prism 10.2 via two-way ANOVA tests (followed by Tukey post-tests for multiple comparisons); statistical significance was considered for *p*-values < 0.05.

## 3. Results and Discussion

**Figure 2:**
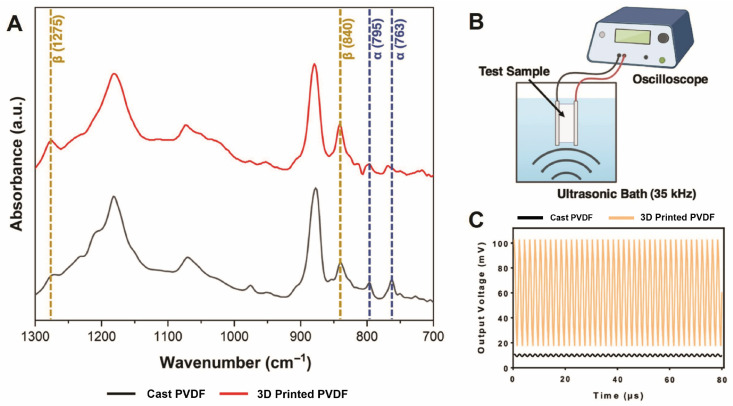
(**A**) FTIR spectra of printed and cast PVDF films for phase characterization. (**B**) Setup to measure output voltage of printed and cast PVDF films using an ultrasonic water bath and oscilloscope. (**C**) Steady-state open-circuit output voltage from PVDF sensors under applied 35 kHz ultrasound over 80 µs. A vertical offset was applied to the voltage outputs for both cast and printed PVDF films for clarity.

In this study, we used a simple ambient wet mixing/pneumatic 3D-printing approach to produce piezoelectric scaffolds with improved biological and electrical properties (Figure 1). A solvent-exchange-assisted printing technique was employed to further enhance the piezoelectric properties of the printed scaffolds. This method performs two essential functions: (1) preventing the 3D geometries from collapsing during drying and (2) further inducing high β-phase in the printed PVDF. Phase characterization of 3D-printed PVDF films was conducted using FTIR spectroscopy. FTIR spectra of the printed PVDF using this solvent-exchange-assisted method and solvent-cast PVDF film is shown in Figure 2A. Unlike the cast PVDF film, which has a strong α-phase absorption peak around 763 cm^−1^, the spectra of printed PVDF show an evident β-phase peak at 1275 cm^−1^ but no clear 763 cm^−1^ α-phase peak [30]. These findings were further evaluated by using a lab-made force sensor to measure the piezoelectric performance (per Section 2.6) of the printed PVDF films. Figure 2B shows the test setup for measuring the output voltage of the force sensors with applied US. Our fabrication approach significantly enhanced the output voltage produced by the sensors with printed PVDF when compared to cast PVDF films (non-piezoelectric) (Figure 2C and Figure S2). Therefore, we concluded that this method for producing scaffolds via pneumatic printing was sufficient for generating piezoelectric, self-powered scaffolds.

**Figure 3:**
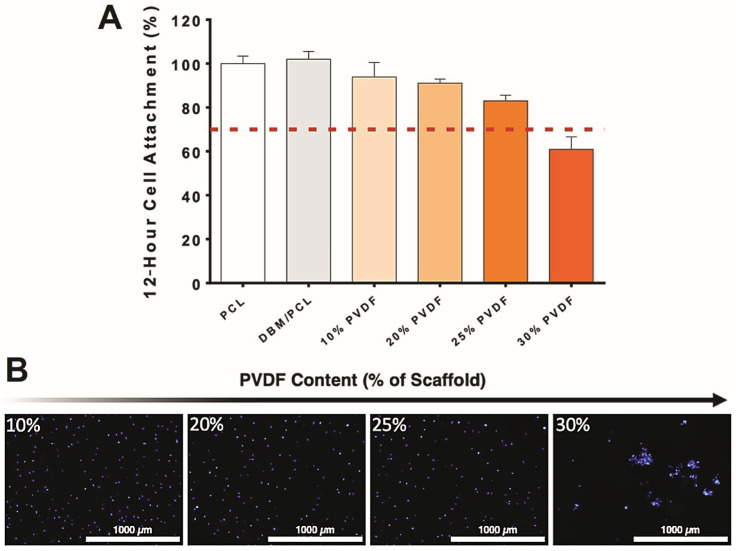
(**A**) Quantitative and (**B**) qualitative 12 h attachment of hMSCs seeded on various PVDF-containing scaffolds. For the quantitative analysis, the red dashed line indicates 70% attachment relative to the PCL-only control group. For the qualitative analysis, cell nuclei were marked using a Hoechst 33342 stain to visualize cell spreading/clustering on scaffold surfaces.

Biomimetic scaffolds for bone tissue engineering should fulfill specific requirements concerning structural and biological characteristics. Therefore, piezoelectric scaffolds based on PVDF are on the forefront for scaffold development, primarily due to their inherent ability to generate surface charges under minor mechanical deformations and their decent mechanical properties. Nevertheless, PVDF is limited by its hydrophobicity, hindering sufficient cellular attachment and expansion, which are essential for building biomimetic bone scaffolds [23]. We assessed the hydrophilicity of our developed composites (based on their PVDF content) using a 12 h cell attachment study. Scaffolds with varying concentrations of PCL and PVDF (DBM content was consistent at 50%) were prepared and pre-wetted ON in growth medium to slightly encourage cellular attachment while allowing the negative effects of excessive PVDF content to be displayed. After a 12 h incubation period, the relative attachment (compared to PCL-only scaffolds) was measured using a CCK-8 assay (Figure 3A). Results show that by incorporating DBM, the attachment of hMSCs slightly increased. This was expected as the addition of a biological scaffolding component, particularly in the form of micro-particles (i.e., improved surface properties), would improve cellular adhesion. Further, our results indicate that as PVDF content increases, the attachment potential of the scaffolds decreases. At 30% PVDF (total wt% of composite scaffold), the hydrophobic nature of the polymer hinders the attachment of seeded cells. In addition to quantitative measurements of cell attachment, qualitative analysis using nuclei staining (Figure 3B) supports these findings. Typical cell spreading can be seen in all PVDF-containing scaffold groups up to 30%.

After confirming the feasibility of producing piezoelectric scaffolds via pneumatic 3D printing and determining an optimal PVDF content for in vitro cell culture, composite scaffolds (50% DBM, 25% PCL, and 25% PVDF) were prepared as described in the Materials and Methods. The printed, and subsequently PDA-coated scaffolds were then gauged for wettability using static water contact angle measurements. Improvements to surface wettability often correlate with enhanced cellular adhesion. That is, surfaces with more moderate hydrophilicity typically improve cell growth compared to those with exceptionally hydrophobic (θ > 150°) or hydrophilic (θ < 5°) surfaces [31]. Water contact angle analysis demonstrated the hydrophilicity of the printed scaffolds before and after the PDA coating (Figure S3). Uncoated PCL, DBM/PCL, and Piezo. DBM/PCL displayed water contact angle values of 74°, 69.4°, and 82.8°, respectively. Scaffolds containing PVDF were more hydrophobic in comparison to the other compositions, as expected. After PDA coating, a significant decrease in water contact angle was observed for all scaffold types, indicating greater surface wettability and subsequently, improved cell affinity potential to the printed scaffolds.

**Figure 4:**
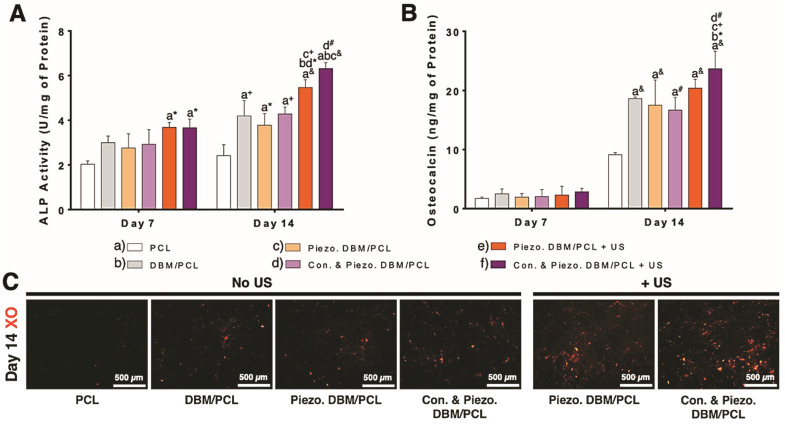
Enhanced osteogenic differentiation observed by combining conductive/piezoelectric scaffolds with ultrasound stimulation (US). (**A**) Early and (**B**) late-stage osteogenic marker expression. (**C**) Xylenol orange, a fluorescent mineral stain of calcified nodules (shown in red color) within cultures either with or without US after 14 days. Here, “Piezo.” refers composites containing PVDF and “Con.” refers to composites coated with a conductive polymer coating. Differences between groups represented by corresponding letters and significance indicated by symbols (* *p* < 0.05; ^+^ *p* < 0.01; ^#^ *p* < 0.001; and ^&^ *p* < 0.0001).

The low frequency US (35 kHz) used in this study has positive correlations to clinical use. It has been shown that lower frequencies are absorbed less by the body, thus limiting any potential damage to the surrounding healthy tissues while allowing for deeper stimulus penetration [32,33]. Our work combines the use of low frequency ultrasound, conductive/piezoelectric biomaterials, and ES to produce a synergistic regenerative effect. The influence of our biomimetic dual-sensing scaffolds combined with US on osteogenic differentiation of hMSCs was investigated after 14 days and compared to cells grown on various scaffolding constructs (with and without US) using both quantitative and qualitative techniques. US consisted of 20 min of 35 kHz stimulation every day for up to 14 days. Cells grown on conductive piezoelectric scaffolds and exposed to US had significantly higher expressions of ALP (Figure 4A) and osteocalcin (Figure 4B) after 14 days, when compared to PCL controls and non-conductive composite scaffolds. There was mineralization present within all groups, as expected after 14 days in osteogenic differentiation medium; however, there were distinctively more deep, red-stained nodules present in the piezoelectric scaffolding groups exposed to US, as shown in Figure 4C. Interestingly, the unstimulated scaffolding groups containing DBM also produced more stained mineralized nodules when compared to the PCL control, further indicating the added biological benefits of incorporating allograft DBM directly into the scaffolding matrix during fabrication. Our in vitro results clearly illustrate that scaffolds possessing conductive/piezoelectric properties and exposed to US strongly induce mineral formation. These results support the use of bone scaffolds based on biomimetic composition in conjunction with US as a tool for enhancing osteogenic differentiation.

## 4. Conclusions

Herein, we demonstrate, for the first time, a simple approach for generating bioactive conductive/piezoelectric scaffolds with controllable architectures appropriate for bone tissue engineering applications. The prepared scaffolds demonstrated suitable piezoelectric properties and prolonged bioactivity through our chosen fabrication method, pneumatic 3D printing. These composites also possess heightened electrical properties through the addition of a conductive coating. Under dynamic culturing conditions (i.e., US), the fabricated scaffolds generated a greater osteogenic response when compared to unstimulated control scaffolds (with and without DBM) after two weeks. Despite the significant advantages of the prepared scaffolds for potential bone regeneration, further studies should be conducted in order to optimize the scaffold formulation (i.e., PVDF and DBM concentrations) and stimulation regimen. For example, the piezoelectric properties of the scaffold could be improved without hindering the biological performance of the scaffolds through the incorporation of other piezoelectric components like zinc oxide, which exhibits low toxicity with small quantities [34,35]. Additionally, simulated in vivo (e.g., bioreactors mimicking physiologic conditions) studies could be performed to further validate scaffold function prior to animal models. Furthermore, a thorough examination of the mechanical and degradative properties of these composites will also need to be conducted. Nonetheless, our preliminary findings present a practical approach for generating biomimetic bone scaffolds that act as self-powered electrical stimulators. The scaffolds developed herein could potentially be used as a basis for developing tissue-like replacements for bone using synthetic biomaterials.

## Data Availability

The original contributions presented in this study are included in the article/Supplementary Material. Due to a fire that affected laboratory data storage, the original microscopy datasets underlying Figure 3B and Figure 4C could not be retrieved. The highest-resolution processed images available have been provided in the manuscript to support the reported findings. Further inquiries can be directed to the corresponding author.

## Supplementary Materials

The following supporting information can be downloaded at: https://www.mdpi.com/article/10.3390/biomimetics10090598/s1. Detailed methods for osteogenic protein assays. Figure S1: Total protein concentrations. Figure S2: Output voltages of PVDF films. Figure S3: Water contact angle measurements.
