# Supplementary material for: Biomimetic Dual-Sensing Bone Scaffolds: Characterization and In Vitro Evaluation Under Dynamic Culturing Conditions
Source: Biomimetics (Basel). 2025 Sep 8;10(9):598. doi: 10.3390/biomimetics10090598 (PMC12467671; doi:10.3390/biomimetics10090598)
Supplement: Supplementary file 1 [file biomimetics-10-00598-s001.zip › biomimetics-3774715-supplementary.pdf]

Supporting Information

Biomimetic Dual Sensing Bone Scaffolds: Characterization and In Vitro Evaluation Under Dynamic Culturing Conditions

Damion T. Dixon[1], Erika N. Landree[2], and Cheryl T. Gomillion[2,]*

[1]School of Environmental, Civil, Agricultural and Mechanical Engineering, College of Engineering, University of Georgia, Athens, Georgia 30602, United States

[2]School of Chemical, Materials and Biomedical Engineering, College of Engineering, University of Georgia, Athens, Georgia 30602, United States

Corresponding Author:

Prof. Cheryl T. Gomillion, Ph.D.

Email: ctgomillion@uga.edu

1. Detailed Methods for Osteogenic Assays

1.1 Alkaline Phosphate Activity

An Alkaline Phosphatase (ALP) Assay Kit (abcam, Waltham, MA, USA) was used test for the presence of osteoblast-like cells over the course of the 14-day long culture period. This assay employs p-nitrophenyl phosphate (pNPP) hydrolyzed by ALP to induce a color change (yellow-colored) related to ALP levels within prepared samples. For the assay, a volume of 20 µL of each protein sample or 120 µL of each standard were pipetted into individual wells in a 96-well plate. A volume of 60 µL of the assay buffer was added to sample wells to bring the total volume up to 80 µL. Subsequently, 50 µL of prepared 5 mM pNPP solution was added to sample wells. The conversion of ALP within the standards was activated with 10 µL of the reconstituted ALP enzyme, and the plate was incubated at room temperature (RT) in the dark for ~1 h. The conversion was stopped with the addition of 20 µL of the stop solution and the absorbance measured at 405 nm. The resulting values were normalized to total protein concentrations for each scaffold type and stimulation group.

1.2 Osteocalcin ELISA Assay

A Human Osteocalcin ELISA Kit (#EKU06413, Biomatik, Wilmington, DE, USA) was used to measure the expression of osteocalcin, a protein preferably secreted by osteoblasts, as directed by the manufacturer's protocol. For the assay, 100 µL of the prepared standards and experimental protein samples were pipetted into a pre-coated 96-well strip plate and placed into an incubator at 37 °C for ~1 h. Next, the protein samples and standards were replaced with 100 µL of reagent A and the plate was placed back in the incubator for ~1 h. Following this incubation step, the standards/samples were removed, the plate was washed with a diluted wash buffer, and 100 µL of reagent B was added before an additional 30 min incubation at 37 °C. Then, after completing a second removal/wash, 90 µL of substrate solution was added, and the plate was incubated for a final time (~20 min at 37 °C). After ending the conversion (with 50 µL of the stop solution), a final color change was produced and the absorbance was read at 450 nm. Absorbance values were normalized as described previously (i.e., utilizing total protein concentrations for each scaffold type or stimulation group).

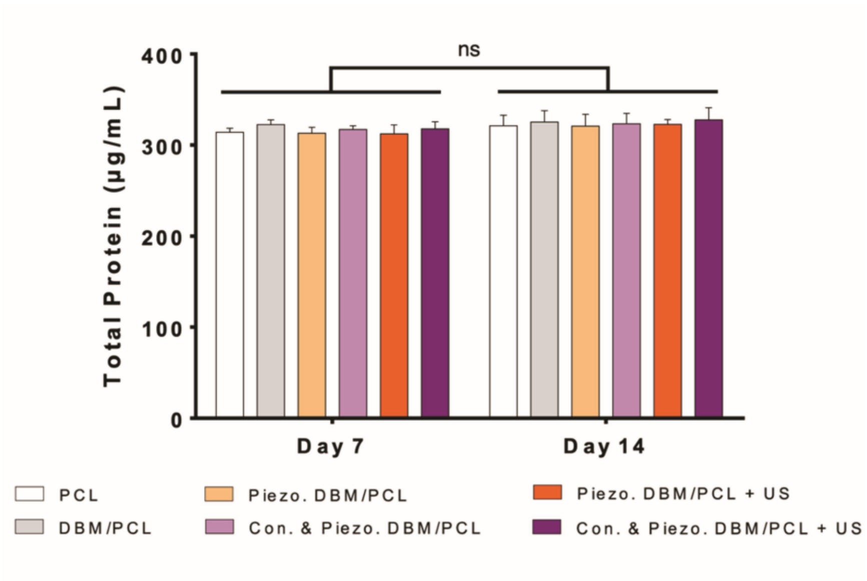

Figure S1. Total protein concentrations over the 14-day culture shown by BCA assay. Here, “Piezo.” refers composites containing PVDF and “Con.” refers to composites coated with a conductive polymer coating. No differences were observed among any groups during culture (i.e., there was no loss in cell viability due to the US treatment).

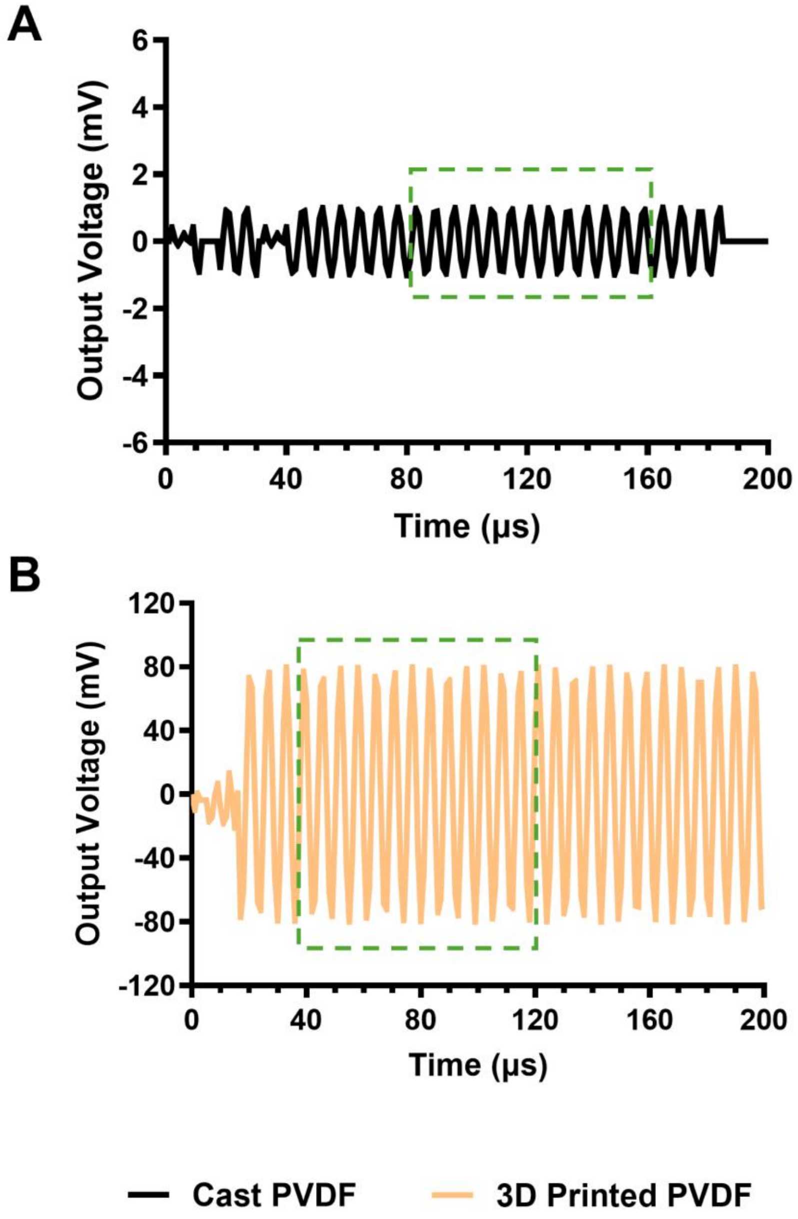

Figure S2. Measurement of open circuit output voltages produced by (A) cast and (B) 3D printed PVDF film sensors under applied 35 kHz ultrasound over 200 μs. Inserts correspond to outputs reported in Figure 2C.

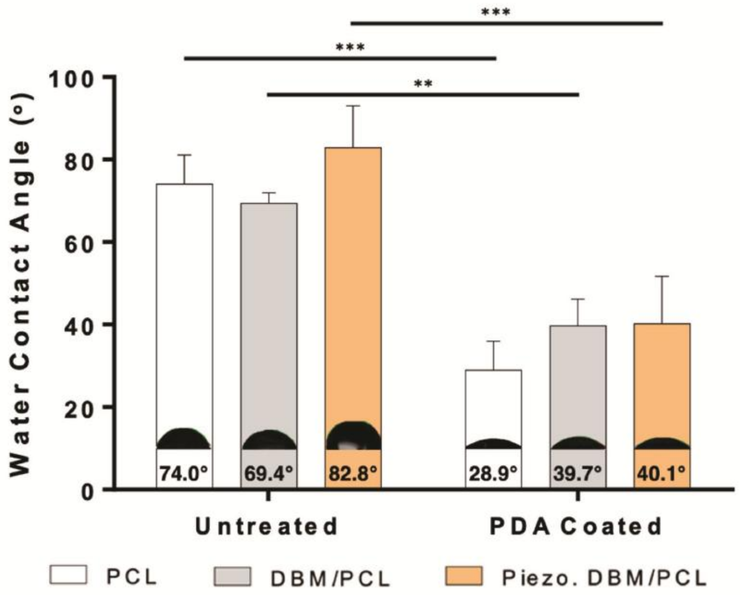

Figure S3. Water contact angle measurements of untreated and PDA coated scaffolds. Here, "Piezo." refers composites containing PVDF. Differences between untreated and PDA coated scaffolds represented by asterisks (** $p < 0.01$; *** $p < 0.001$).
